# Chromosome-level genome assembly and annotation of the maize weevil (*Sitophilus zeamais* Motschulsky)

**DOI:** 10.1038/s41597-025-05341-w

**Published:** 2025-06-09

**Authors:** Yueliang Bai, Fangfang Zeng, Meng Zhang, Chao Zhao, Shuai Pang, Guiyao Wang

**Affiliations:** 1 Henan Collaborative Innovation Center for Grain Storage Security, School of Food and Strategic Reserves, Henan University of Technology, Zhengzhou, China; 2 Glbizzia Biosciences, Beijing, China; 3 Zhengzhou Tobacco Research Institute of CNTC, Zhengzhou, China

**Keywords:** Agricultural genetics, DNA sequencing

## Abstract

The maize weevil, *Sitophilus zeamais* Motschulsky, is one of the most destructive pests of stored grains worldwide, posing a significant threat to global food security. To better understand the biology, resistance mechanism, and adaptive evolution of this species, we presented a high-quality chromosome-level genome assembly of *S. zeamais* using PacBio sequencing and Hi-C technologies. The size of the final assembled genome was 693.21 Mb with scaffold N50 of 61.03 Mb, and 631.97 Mb were successfully anchored into 11 pseudochromosomes. In total, 15,161 protein-coding genes were annotated, of which 98.89% obtained functional descriptions. Additionally, 377.50 Mb of sequences were identified as repeat elements, accounting for 54.46% of the genome. BUSCO analysis revealed a high level of completeness in both the genome assembly and annotation, with scores of 98.17% and 97.22%, respectively. The chromosome-level genome of *S. zeamais* provides valuable genomic insights that deepen our understanding of the evolution and ecology of *Sitophilus* species, while also contributing to the development of targeted and innovative control strategies for stored-product pests.

## Background & Summary

**Fig. 1:**
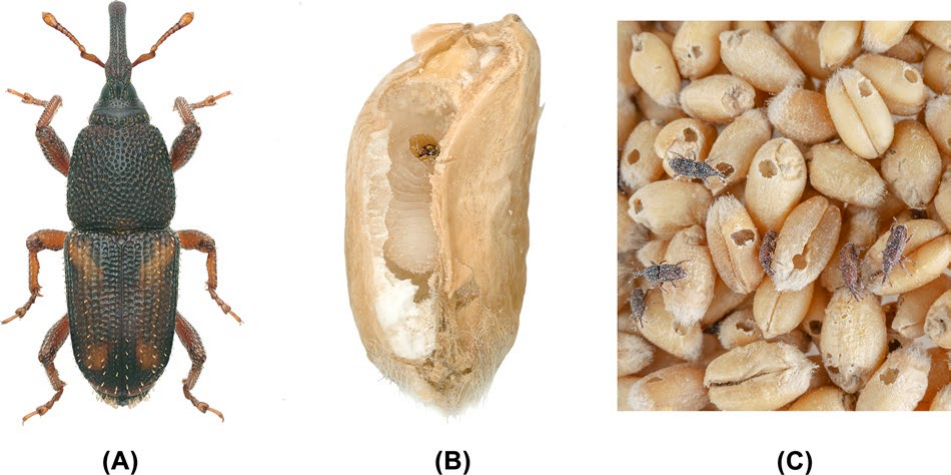
Photos of *S. zeamais*. (**A**) A *S. zeamais* adult. (**B**) A *S. zeamais* larva inside a wheat kernel. (**C**) Infestation of *S. zeamais* on wheat.

The maize weevil, *Sitophilus zeamais* Motschulsky, is one of the most destructive pests of stored grains, especially maize, in many countries or regions, causing severe economic losses each year^1–4^. Studies have demonstrated that *S. zeamais* damages a wide range of cereals, including staple grains such as maize, wheat, and rice^5–7^, posing a serious threat to the global food security. As a primary pest, maize weevils can infest grains before or after harvest^8,9^, and cause direct damage to grains through feeding and oviposition during the storage period, leading to severe quantitative and qualitative losses^10–12^. In addition, their activities increase the moisture and temperature in the grain mass, indirectly promoting the occurrence and development of the secondary pests and microorganisms, further exacerbating the severity of the damage^13,14^. Moreover, the immature stages of the weevil develop inside grains^15^, where they are well protected and difficult to detect, which is one of the key reasons for their frequent and severe outbreaks (Fig. 1).

Currently, the genomic resources available for stored product pests are still very limited compared to those for the field and public health pests. Species of *Sitophilus* are among the most evolutionarily successful pests in stored grains, including several important stored-product pests such as *S. zeamais*, *S. oryzae*, *S. granaries*, and others^14,16^. For now, only one scaffold fragmented genome (*S. oryzae*) from *Sitophilus* genus has been published^17^, and the lack of high-quality chromosome-scale genome assembly limits in-depth investigations of structural variations^18^, gene linkages^19^, and regulatory regions^20^, which are crucial for understanding the adaptive evolution of these species in stored-product environments. Furthermore, the differences in cold tolerance^21^, food preferences^22^, and geographic distribution^3^ among *Sitophilus* species highlight the need for more genomic resources to uncover the genetic mechanisms behind their adaptive divergence, which is crucial for ensuring grain storage security across different regions and cereal types.

In this study, a high-quality chromosome-level genome assembly of *S. zeamais* was obtained through a combination of PacBio high-fidelity (HiFi) sequencing and chromosome conformation capture (Hi-C) sequencing technologies. The size of the final assembled genome was 693.21 Mb with scaffold N50 of 61.03 Mb, and 91.17% of the bases were successfully clustered and ordered into 11 pseudochromosomes. Through genome annotation, 54.46% of the genome was annotated as repeat sequences, and 15,161 protein-coding genes were obtained by structural annotation, of which 98.89% were functionally described. In summary, the chromosome-scale genome assembly of *S. zeamais* would not only deepen our understanding of the adaptive evolution of *Sitophilus* species, but also contribute to the development of targeted and novel control strategies for stored-product pests.

## Methods

### Insects

The adults of *S. zeamais* were initially collected from a grain storage facility in Zhengzhou, Henan Province, China. Approximately ten pairs of adults were isolated from the collected infested wheat under a microscope, with each pair reared separately in an individual container. The population was then continuously reared with intact wheat grains for over 20 generations at 30 ± 1 °C, 70% ± 5% relative humidity, and a 24-hour dark photoperiod. All individuals used for sequencing in this study were derived from the offspring of one of the original pairs.

### Library construction and sequencing

For whole genome sequencing, Pacbio HiFi library was constructed from a single male adult of *S. zeamais* with an ultra-low input DNA library construction method. Firstly, genomic DNA was extracted using the FineOut Universal Genomic DNA Extraction Kit (Genfine, China), and the integrity was assessed by Femto Pulse (Agilent Technologies, USA). The obtained DNA (0.38 ug) was fragmented by the Megaruptor^®^ 3 system (Diagenode, Belgium) and purified using AMPure PB magnetic beads (Beckman, USA). Then, two rounds of PCR were performed to amplify the purified DNA. Next, the amplified DNA was used to construct the SMRTbell library with the SMRTbell Express Template Prep Kit 2.0 (Pacific Biosciences, USA) with an insert size of 10 kb. Finally, sequencing was conducted on the Pacbio Revio platform, and a total of 46.07 Gb HiFi clean reads were generated (Table 1). For Hi-C sequencing, 180 adult male *S. zeamais* individuals were first crosslinked with 2% formaldehyde, followed by DNA digestion with *Mbol* restriction endonuclease for library construction. The resulting DNA fragments were then sequenced on the Illumina Novaseq X plus platform with PE150 strategy, yielding 69.39 Gb of Hi-C clean reads (Table 1).

**Table 1:** Statistics of sequencing data, genome assembly, and genome annotation of *S. zeamais*.

Item	Features		Statistics
Sequencing data	Genome	Pacbio HiFi data (Gb)	46.07
		Illumina Hi-C data (Gb)	69.39
	Transcriptome	Illumina RNA-Seq data (Gb)	55.67
		Pacbio Iso-Seq data (Gb)	9.70
Assembly	Genome size (Mb)		693.21
	Scaffold N50 (Mb)		61.03
	Karyotype		2n = 22
	Bases anchored to chromosomes (%)		91.17
	GC content (%)		32.15
	BUSCO complete of the genome assembly (%)		98.17
Annotation	Repeat elements (%)		54.46
	Number of PCGs		15,161
	Number of non-coding RNAs		1,411
	BUSCO complete of PCGs (%)		97.22

For transcriptome sequencing, 30–50 first- to third-instar larvae, 20–30 fourth-instar larvae, prepupa, pupa, male adults, and female adults were collected for RNA isolation. Invitrogen TRIzol Reagent (Thermo Fisher Scientific, USA) was used to extract RNA from the above samples according to the manufacturer’s protocol. After assessing RNA purity and integrity, eight conventional transcriptome libraries were constructed from samples of different development stages or sex, and sequenced on the Illumina Novaseq X plus platform (Table S1). For full-length transcriptome sequencing, equal amounts of total RNA from each of the eight developmental stages or sexes were pooled, and a total of 300 ng RNA was used for library construction, which was subsequently sequenced on the Pacbio Revio platform (Table 1). After quality control, a total of 55.67 Gb and 9.70 Gb filtered clean data was generated from the Illumina and Pacbio platforms, respectively.

### Genome feature estimation

**Fig. 2:**
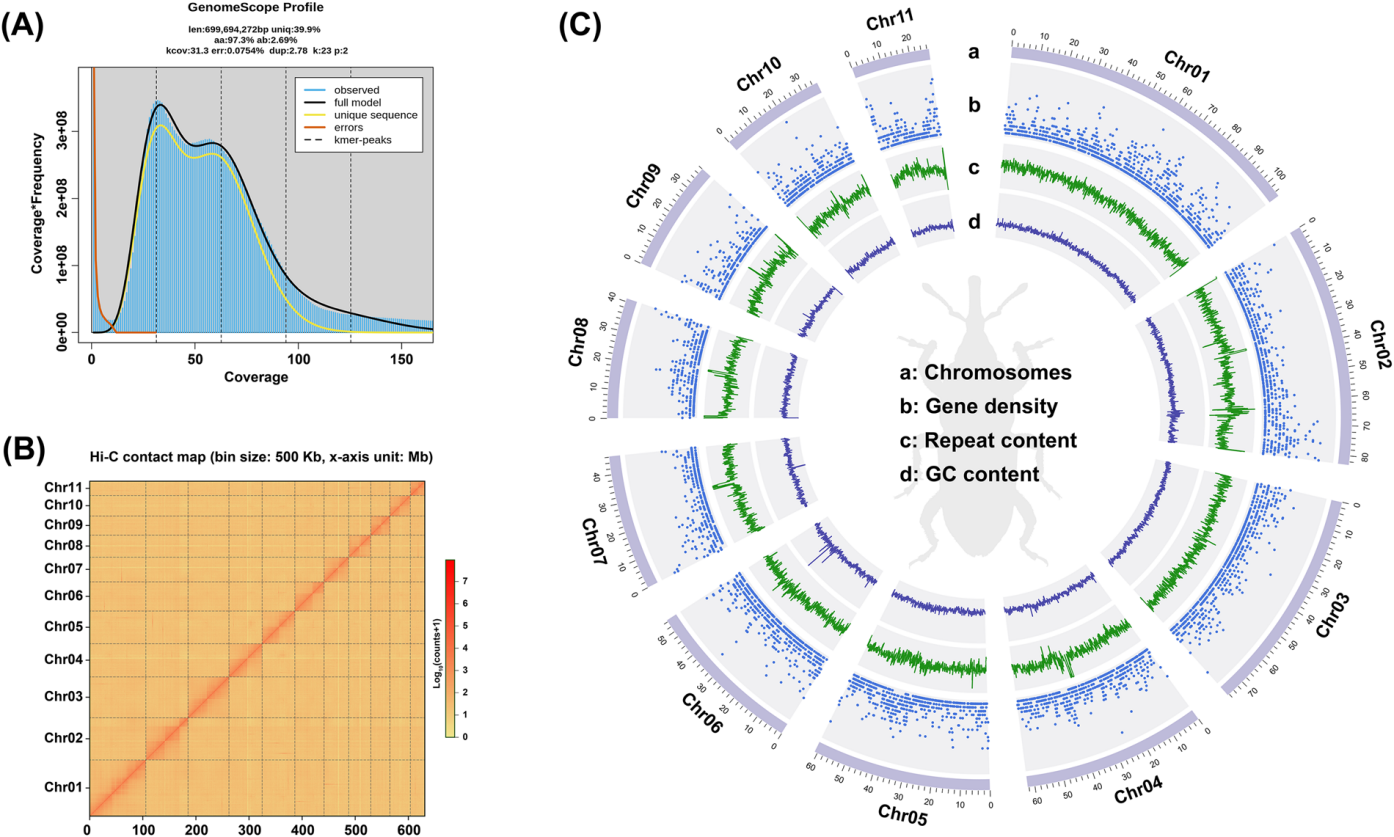
Genome assembly of *S. zeamais*. (**A**) Estimation of genome features based on k-mer analysis (k = 23). (**B**) Genome-wide Hi-C interactions among 11 pseudochromosomes of *S. zeamais*. (**C**) Genome landscape of *S. zeamais* (scale is in Mb): (a) Length of pseudochromosomes; (b) Density of gene numbers; (c) Content of repeat sequences; (d) GC content.

To estimate the genome features of *S. zeamais*, we performed a genome survey using Pacbio data. The genome size of *S. zeamais* was estimated to be 699.69 Mb based on K-mer analysis (K = 23) conducted with Jellyfish v2.3.1^23^ and GenomeScope v2.0^24^. Additionally, the genome exhibited a high heterozygous ratio of 2.69% and a high repeat rate of 60.10% (Fig. 2A).

### Genome assembly

For genome assembly, HiFi reads from Pacbio platform were first assembled into contigs using Hifiasm v0.19.1-r559^25^ with default parameters. The assembled draft genome size was 693.11 Mb with a contig N50 of 1.25 Mb. Then, clean reads from the Hi-C library were aligned to the draft assembly by HICUP v0.8.2^26^. Chromosome-level scaffolds were then constructed, sealed, and merged using ALLHiC v0.9.8^27^. The final placement and orientation of scaffolds on chromosomal groups were determined based on interaction signals using Juicebox v1.11.08^28^ (Fig. 2B, Table S2). Ultimately, a chromosome-scale genome assembly of 693.21 Mb with a scaffold N50 of 61.03 Mb was achieved, with 91.17% of the bases successfully anchored into 11 pseudochromosomes (Fig. 2C, Table S3). Benchmarking Universal Single-Copy Orthologs (BUSCO) v5.5.0^29^ was used to evaluate the completeness of the genome assembly, resulting in a high BUSCO score of 98.17% with insecta_odb10 database (Table S4).

### Repeat annotation

**Fig. 3:**
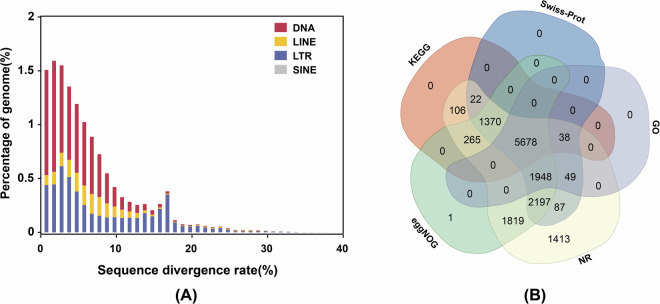
Genome annotation of *S. zeamais*. (**A**) Distribution of sequence divergence rates across different types of annotated repetitive elements. (**B**) Functional annotation of protein-coding genes in five databases, including Swiss-Prot, GO, NR, eggNOG, and KEGG.

Repetitive element annotation was performed using both homology-based and *de novo* methods. For the homology-based annotation, LTRharvest v1.6.2^30^, LTR_Finder v1.07^31^, and LTR_retriever v2.9.0^32^ were used to construct a non-redundant LTR-RT library with default parameters. MITE-Hunter v1.0.0^33^ was used to identify the miniature inverted-repeat transposable elements (MITEs). For *de novo* prediction, RepeatModeler v2.0.2a^34^ was used to generate a *de novo* repeat library. Finally, RepeatMasker v4.1.2^35^ was used to predict repeat sequences in the genome by searching against the combination of the constructed libraries. As a result, a total of 377.50 Mb sequences were identified as repeat elements, constituting 54.46% of the *S. zeamais* genome (Table 2, Fig. 3A).

**Table 2:** Statistics of annotated repeat elements in the genome of *S. zeamais*.

Repeat type	Number of elements	Length (bp)	Percent
**Retrotransposons**	**340,123**	**146,559,023**	**21.14%**
LTR/Copia	1,501	563,679	0.08%
LTR/DIRS	4,23	191,890	0.03%
LTR/Gypsy	39,228	26,651,806	3.84%
LTR/others	230,292	83,452,361	12.04%
LINE	68,668	35,685,314	5.15%
SINE	11	13,973	0.00%
**DNA Transposons**	**256,809**	**140,623,384**	**20.29%**
DNA/Academ	1,765	1,122,206	0.16%
DNA/CMC	283	135,749	0.02%
DNA/Crypton	8,657	3,820,023	0.55%
DNA/Maverick	2,570	4,036,742	0.58%
DNA/PIF	1,005	609,587	0.09%
DNA/PiggyBac	218	82,268	0.01%
DNA/Sola	184	82,265	0.01%
DNA/TcMar	5,831	3,587,957	0.52%
DNA/hAT	4,620	3,326,630	0.48%
Others	231,676	123,819,957	17.62%
**Rolling-circles**	**26**	**3,425**	**0.00%**
**Simple repeats**	**5,046**	**2,314,527**	**0.33%**
**Unknown**	**98,714**	**88,000,886**	**12.69%**
**Total**	**700,718**	**377,501,245**	**54.46%**

Note: TE, transposable element; LINE, long interspersed nuclear elements; SINE, short interspersed nuclear elements; LTR, long terminal repeats.

### Protein-coding genes annotation

After masking repeat sequences, the genome assembly was used for protein-coding genes (PCGs) annotation by integrating three types of evidence: *de novo*, homology, and transcript prediction. For *de novo* annotation, Augustus v3.2.2^36^, GlimmerHMM v3.0.4c^37^, and GeneMark-ET v1.0^38^ were used to predict the genome structure based on the statistical characteristics of genomic sequence data. For homology prediction, protein sequences of five species, including *S. oryzae*, *Rhynchophorus ferrugineus*, *Dendroctonus ponderosae*, *Tribolium castaneum*, and *Drosophila melanogaster*, were aligned to the genome assembly with MMseqs v2.0^39^ and predicted by GeMoMa v1.6.1^40^. For the transcriptome-based strategy, conventional and full-length transcriptome data were mapped to the genome by HISAT2 v2.1.0^41^ and PASA v2.1^42^. Finally, EvidenceModeler v1.1.1^43^ was employed to generate a complete non-redundant gene set by integrating three types of evidence. The completeness of the final gene set was assessed to be 97.22% by BUSCO analysis (Table S5). For function annotation, the predicted PCGs were aligned to Swiss-Prot, NCBI Non-Redundant (NR), and eggNOGv5 databases by diamond v2.0.13^44^, and to Gene Ontology (GO), and Kyoto Encyclopedia of Genes and Genomes database (KEGG) databases using KOBAS v3.0^45^ (Fig. 3B). Overall, a total of 15,161 genes were annotated as PCGs, among which 14,993 genes (98.89%) obtained functional descriptions from at least one database (Table 3).

**Table 3:** Statistics of predicted protein-coding genes in the genome of *S. zeamais*.

Property	Value
Number of genes	15,161
Total genic length (bp)	286,189,668
Mean gene length (bp)	18,876
Number of transcripts	21,108
Transcripts per gene	1.4
Total transcript length (bp)	49,610,238
Mean transcript length (bp)	2,350
Number of exons	151,101
Exons per transcript	7.2
Mean exon length	328
Number of coding exons	143,624
Number of introns	129,993
Mean intron length (bp)	3115
Total CDS length	35,201,333
Mean CDS length	1,667

### Non-coding RNA annotation

Non-coding RNA was predicted by two strategies. Firstly, tRNA sequences were predicted using the software tRNAscan-SE v1.3.1^46^. Then, rRNA, snRNA, and miRNA were identified by searching against the Rfam database^47^ with infernal v1.1.4^48^. Finally, a total of 1,411 sequences were predicted as non-coding RNA, accounting 0.03% of the genome (194.59 kb), and included 57 miRNA, 219 tRNA, 1,118 rRNA, and 17 snRNA (Table 4).

**Table 4:** Statistics of predicted non-coding RNAs in the genome of *S. zeamais*.

Type	Number	Total length (bp)	Percentage (%)
miRNA	57	4,813	0.0007
tRNA	219	16,223	0.0024
rRNA	1,118	171,303	0.0252
snRNA	17	2,254	0.0003
Total	1,411	194,593	0.0287

## Data Records

All the raw sequencing data of *S. zeamais* have been uploaded to the NCBI BioProject database under PRJNA1208214^49^. The genomic Pacbio sequencing data (SRR32211809^50^), Hi-C sequencing data (SRR31950408^51^), Illumina RNA-Seq data (SRR31951228-SRR31951235^52–59^) and Pacbio Iso-Seq data (SRR31950843^60^) were available under the Sequence Read Archive (SRA) database. The genome assembly was deposited at GenBank with the accession number JBLKPX000000000^61^. Besides, the genome assembly and annotation file were also available in Figshare^62^.

## Technical Validation

### Validation of genome assembly

BUSCO v5.5.0^29^ was used to evaluate the completeness and contiguity of the genome assembly with insecta_odb10 database. The analysis indicated that 98.17% of the conversed insect orthologues (single-copy genes: 97.44%, duplicated genes: 0.73%) were completely captured by the chromosome-level genome assembly of *S. zeamais* (Table S4).

### Validation of genome annotation

BUSCO v5.5.0 was also used to assess the genome annotation with insecta_odb10 database, which showed a high completeness with 97.22% BUSCO genes (single-copy genes: 95.90%, duplicated genes: 1.32%) were captured by the PCGs of *S. zeamais* (Table S5).

## Supplementary information

Supplemental Information

## Data Availability

All analyses were performed following the manuals and protocols of the cited bioinformatic software without special codes.
