# Supplementary material for: Chromosome-level genome assembly and annotation of the maize weevil (Sitophilus zeamais Motschulsky)
Source: Sci Data. 2025 Jun 9;12:966. doi: 10.1038/s41597-025-05341-w (PMC12149295; doi:10.1038/s41597-025-05341-w)
Supplement: Supplementary file 1 — Supplemental Information [file 41597_2025_5341_MOESM1_ESM.docx]

**Supplemental Information for:**

**Chromosome-level genome assembly and annotation of the maize weevil (*Sitophilus zeamais* Motschulsky)**

Yueliang Bai^1*^, Fangfang Zeng^1^, Meng Zhang^1^, Chao Zhao^1^, Shuai Pang^2^, Guiyao Wang^3^

**Table of contents:**

| **Title and authors** | **Page1** |
| --- | --- |
| **Table S1** | **Page 2** |
| **Table S2** | **Page 3** |
| **Table S3** | **Page 4** |
| **Table S4** | **Page 5** |
| **Table S5** | **Page 6** |

**Table S1. Summary of RNA-Seq data in this study**

| **Sample** | **Clean reads** | **Clean bases** | **Q30 (%)** |
| --- | --- | --- | --- |
| 1st instar larva | 49,518,022 | 7,427,703,300 | 97.56 |
| 2nd instar larva | 38,944,500 | 5,841,675,000 | 97.92 |
| 3rd instar larva | 41,770,966 | 6,265,644,900 | 97.99 |
| 4th instar larva | 48,273,086 | 7,240,962,900 | 97.75 |
| Prepupa | 43,400,976 | 6,510,146,400 | 98.29 |
| Pupa | 48,735,964 | 7,310,394,600 | 98.16 |
| Female adult | 53,611,244 | 8,041,686,600 | 98.25 |
| Male adult | 46,898,628 | 7,034,794,200 | 97.97 |

**Table S2. Statistics of anchored Information of pseudochromosomes by Hi-C**

| **Pseudomolecule** | **Length (bp)** | **GC content (%)** |
| --- | --- | --- |
| Chr01 | 105,806,221 | 32.61 |
| Chr02 | 83,128,483 | 32.63 |
| Chr03 | 76,647,778 | 32.67 |
| Chr04 | 62,494,831 | 32.77 |
| Chr05 | 61,034,850 | 32.88 |
| Chr06 | 54,645,904 | 32.85 |
| Chr07 | 46,306,991 | 32.59 |
| Chr08 | 41,132,464 | 32.60 |
| Chr09 | 38,105,225 | 32.84 |
| Chr10 | 35,862,229 | 32.81 |
| Chr11 | 26,802,832 | 32.78 |
| Total anchored | 631,967,808 | 32.61 |
| Unanchored | 61,245,157 | 32.63 |

**Table S3. Statistics of contig- and chromosome-level assembly of *S. zeamais***

| **Items** | **Contig**  **length (bp)** | **Contig**  **number** | **Scaffold**  **length (bp)** | **Scaffold number** |
| --- | --- | --- | --- | --- |
| Total | 693,112,865 | 1,031 | 693,212,965 | 30 |
| Max | 7,262,327 | - | 105,806,221 | - |
| Number≥2000bp | - | 1,031 | - | 30 |
| N50 | 1,251,010 | 175 | 61,034,850 | 5 |
| N60 | 1,036,881 | 235 | 54,645,904 | 6 |
| N70 | 832,965 | 310 | 46,306,991 | 7 |
| N80 | 622,150 | 407 | 38,105,225 | 9 |
| N90 | 373,844 | 547 | 26,802,832 | 11 |

**Table S4. Statistics of BUSCO assessment of *S. zeamais* genome assembly**

| **Type of BUSCOs** | **Number** | **Percentage (%)** |
| --- | --- | --- |
| Complete BUSCOs | 1,339 | 98.17 |
| Complete and single-copy BUSCOs | 1,332 | 97.44 |
| Complete and duplicated BUSCOs | 10 | 0.73 |
| Fragmented BUSCOs | 2 | 0.15 |
| Missing BUSCOs | 22 | 1.68 |
| Total BUSCO groups searched | 1,367 | 100.00 |

**Table S5. Statistics of BUSCO assessment of *S. zeamais* genome annotation**

| **Type of BUSCOs** | **Number** | **Percentage (%)** |
| --- | --- | --- |
| Complete BUSCOs | 1,329 | 97.22 |
| Complete and single-copy BUSCOs | 1,311 | 95.90 |
| Complete and duplicated BUSCOs | 18 | 1.32 |
| Fragmented BUSCOs | 3 | 0.22 |
| Missing BUSCOs | 35 | 2.56 |
| Total BUSCO groups searched | 1,367 | 100.00 |
